# The use of a microcomputer for flexible automation of a liquid chromatograph

**DOI:** 10.1155/S1463924679000419

**Published:** 1979

**Authors:** A. D. Mills, I. Mackenzie, R. J. Dolphin

**Affiliations:** 1 Philips Research Laboratories Surrey Redhill RH1 5HA UK; 2 Pye Unicam Ltd. York Street Cambridge CB1 2PX UK


value (STOP @ ), the two are compared to > to see if the incremented value at LOCATION is larger then the STOP value, if it is the program ends, if not, it repeats the process starting at BEGIN–HERE.

It should be noted that whilst many variables have been pushed on the stack, only the data will remain, since each time a value is used it is 'popped' (removed) from the stack. If a different spectra region is to be scanned i.e. from 3000 to 6500 with 10 increments the variables need only be changed thus

```
3000 START !
6500 STOP  !
  10 INCREMENT !
```

and type SCAN, system will now scan from 3000 to 6500 taking data every 10 steps.

While the code might look a little strange at first, it quickly becomes very easy to work with. The SCAN program of Figure 6 could be combined with other modules as shown in Figure 5 to perform some more complex experimental function. Each module of the program can be easily tried out to ensure that it is operational before proceeding with the next.

Presently, CONVERS is being used in the authors' laboratories for a variety of spectrochemical investigations, including laser excited optoacoustic spectroscopy (Figure 7) and inductively coupled plasma optical emission spectroscopy (Figure 8). Rather complex interactive control and data acquisition programs have been easily implemented. Memory requirements and operating speed have been found to be far superior to conventional approaches. Additionally, new system users have encountered a difficulty in utilising previously developed custom software for a particular experiment even when documentation was vague.

### Discussion

The authors hope that this short introduction to only a few of the concepts employed in CONVERS will generate interest in its capabilities. A much more complete discussion is available in the form of a user's manual [3] available from the authors.

ACKNOWLEDGEMENTS
The development of the CONVERS system was partially supported by the Office of Naval Research and a Alfred P. Cloan Foundation Research Fellowship to M. Bonner Denton.

REFERENCES

[1] C. Moore, *Astronomical Astrophysics Supplemental,* 15,(1974) 497.
[2] M.B. Denton, J.D. Mack, M.W. Routh and D.B. Swartz,*American Laboratory,* 8,69 (1976).
[3] CONVERS An Interpretive Compiler, developed by Scott B. Tilden and M. Bonner Denton, Department of Chemistry, University of Arizona, Tucson, Arizona 85721, USA.

### The use of a microcomputer for flexible automation of a liquid chromatograph

A.D. Mills, I. Mackenzie and R.J. Dolphin*

*Philips Research Laboratories, Redhill, Surrey, RH1 5HA, U.K.*

### Introduction

Microprocessors are being used to add inexpensive automatic control and data handling facilities to a variety of chemical instruments. With a microcomputer it is now possible to realise the flexibility formerly available only with a relatively large and expensive minicomputer in an instrument little different in size and cost from one controlled by inflexible hardware. In many ways chromatography is an ideal process for such automation. Most instruments are given a high workload and, although many applications may be routine and repetitive, the versatility of the technique requires an instrument which can easily be used in a variety of modes.

In addition to improving the convenience to the user, automation of a liquid chromatrograph should enhance the performance of the instrument. Some aspects of high performance liquid chromatography (HPLC) which can benefit in this way are as follows:

(1) Accurate control of solvent flow rate will compensate for changes in pressure drop and lead to more reliable retention times.
(2) The composition of the mobile phase can be accurately controlled in either isocratic or gradient elution chromatography using, for example, a proportioning valve on the low pressure side of the pump.
(3) Automatic sampling can be operated in a variety of modes to process a number of samples without supervision. It is also more precise than manual injection.
(4) A built-in data handling facility can present the analyst with an easily read post-run report of the analytical results with accurate peak area measurements even for peaks which are poorly resolved.

Although liquid chromatographs incorporating microprocessors for control and data handling purposes are commercially available, these instruments are, so far, relatively inflexible. This paper describes, in detail, the automation of a liquid chromatograph using an inexpensive general purpose microcomputer, which has previously been applied in atomic absorption spectrophotometry [1] and for column switching in HPLC [2].

Figure 1 illustrates the interconnection of the chromatograph and the microcomputer which controls the mobile phase flow rate, operates an automatic sampler and analyses data from the detector. The control and data handling functions are integrated in a program which enables the user to communicate with the instrument, in plain English, via a visual display unit (VDU) or teletypewriter keyboard. A variety of operational modes is offered, giving the analyst an opportunity to establish the best conditions for a particular separation before leaving the instrument to perform its given tasks without further interaction.

### The microcomputer

### Hardware

The computer is a general purpose instrument constructed using a set of ready made circuit cards (Philips, Science and

**Present address: Pye Unicam Ltd., York Street, Cambridge, CB1 2PX, U.K.*

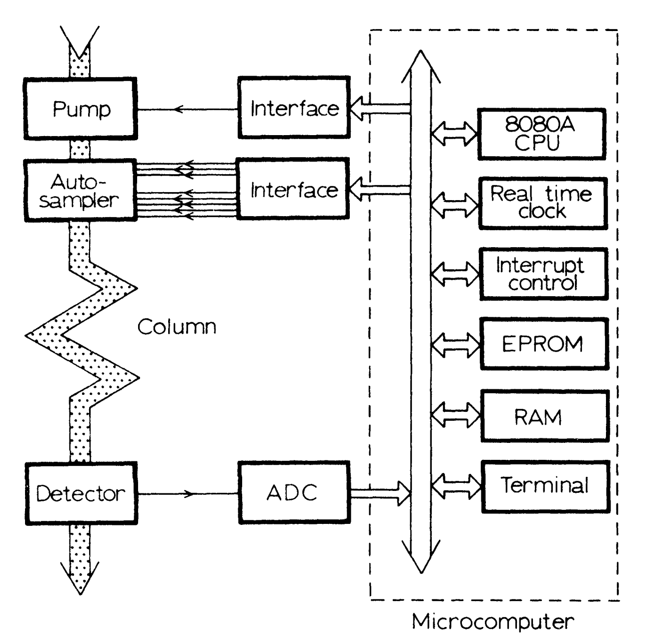

*Figure 1. A schematic of the computer, the chromatograph, and their interfaces.*

Industry Group, Eindhoven, The Netherlands). The central processor is an Intel 8080A. The memory consists of 4K bytes of programmable read-only memory (EPROM) for the system monitor and a number of commonly used subroutines, 16·K bytes of EPROM for developed programs, and 12K bytes of static random access memory (RAM) for storing data or programs under development. Other cards are included for interrupt control, for communication with peripherals, and for a real-time clock. Seven-segment displays on the front panel show elapsed time and chromatographic variables such as flow rate and pressure. Eight light-emitting diodes are also incorporated in the front panel to display the status of programmed variables such as the control byte for the automatic sampler, which will be described later.

### Software

Our laboratories have extensive support for the 8080, including facilities for software development in either assembly language or a high-level language (Coral 66). We can use the multi-access laboratory computer (ICL 1904S) or an Intel MDS microcomputer development system. For the present work, most software development was carried out on the main laboratory computer and output in machine code on paper tape, which was then loaded into RAM using a high-speed paper tape reader. Programs could then be run, debugged, altered etc., using the system monitor resident in EPROM. Some aspects of the control and data handling programs will be described in later sections; in general the control programs were written in assembly language, the data handling program in Coral 66. A 24 bit software floating point mathematics package was used for multiple precision arithmetic. The complete program occupies approximately 8K bytes of memory.

### Pump control

The pump used in this instrument (Pye-Unicam Ltd. model LC3-XP) is a precision metering device with its own sophisticated electronics for constant flow control. The flow rate is normally selected using front panel switches but can be set using a signal to a remote control socket at the rear of the pump. It is this latter option which is used in this application. A purpose built interface converts a 14 bit control word into the appropriate pulse width modulated signal to control the pump. In this way a potential precision of better than 0.001 ml/min can be achieved. The required flow rate can be typed in from the keyboard as a decimal number, or the flow can be programmed to change during a run. A maximum pressure for the system can be selected before a run. In the event of this pressure being exceeded, perhaps because of a blocked column, the pump will be switched off.

### Automatic sampler

The automatic sampling system can handle up to twenty-four samples, each in a glass vial sealed with a bonded silicone/PTFE septum. The vials are contained in a circular rack mounted on a turntable which can be rotated to any position in either direction by a stepper motor (Philips, type 9904 112 04002) controlled by an integrated circuit (SAA 1027).

The sample is introduced at the head of the chromatographic column by a six-port loop valve (Rheodyne Co., model 70–10) rotated by a pneumatic actuator. The sample is transferred from the vial to the loop valve through a concentric needle assembly which is lowered by a syringe actuator, piercing the septum seal, into the sample vial. Compressed air or an inert gas is connected to the outer tube thus pushing some of the sample through the inner needle to the sample valve. Figure 2 is a schematic diagram illustrating these pneumatic operations. Valves $V_0$, $V_1$, $V_2$, $V_3$ and $V_4$ are all operated by 24 V d.c. solenoids and are two-position valves which allow the use of a binary control sequence. $V_0$ controls actuator A, which operates the sampling valve. $V_1$ operates cylinder $A_2$ which moves the needle assembly. $V_3$ allows air to pass into the sample transfer system. $V_2$ will switch this air supply either to the inner needle to purge the sample line or to the outer tube to take up the sample. The flow rate of gas through these paths is controlled by a pressure regulator (Watts Ltd., type 362-1) and a restrictor (400 mm x 0.25 mm i.d.). A volume of 100 ml is included between the pressure regulator and $V_2$ to absorb any surge in pressure which may occur when $V_2$ is switched. $V_4$ is used only when multiple samples are to be

**Table 1. Pneumatic control functions**

| Valve | Controlled function | Not energised (0) | Energised (1) |
|---|---|---|---|
| $V_0$ | Sampling valve | Fill | Inject |
| $V_1$ | Needle assembly | Up | Down |
| $V_2$ | Direction of air flow | Purge | Fill |
| $V_3$ | Air supply | Off | On |
| $V_4$ | Pressure equalisation | Shut | Open |

**Table 2. Control sequence for repetitive sampling**

| | $V_4$ $V_3$ $V_2$ $V_1$ $V_0$ | Duration (s) | Comment |
|---|---|---|---|
| 1 | 0 0 0 1 0 | 15 | Needle enters vial |
| 2 | 0 1 0 1 0 | 5 | Air on, line purged |
| 3 | 0 1 1 1 0 | 4 | Loop filled |
| 4 | 0 0 1 1 0 | 1 | Air off, hold loop full |
| 5 | 1 0 1 1 1 | 5 | Injection with pressure equalisation |
| 6 | 0 0 1 1 0 | 2 | End of injection |
| 7 | 0 1 1 1 0 | 3 | Outer tube vented |
| 8 | 0 1 0 1 0 | 7 | Loop emptied |
| 9 | 1 0 0 1 0 | 2 | Pressure equalised |
| 10 | 0 0 0 1 0 | 2 | Rest then return to start |

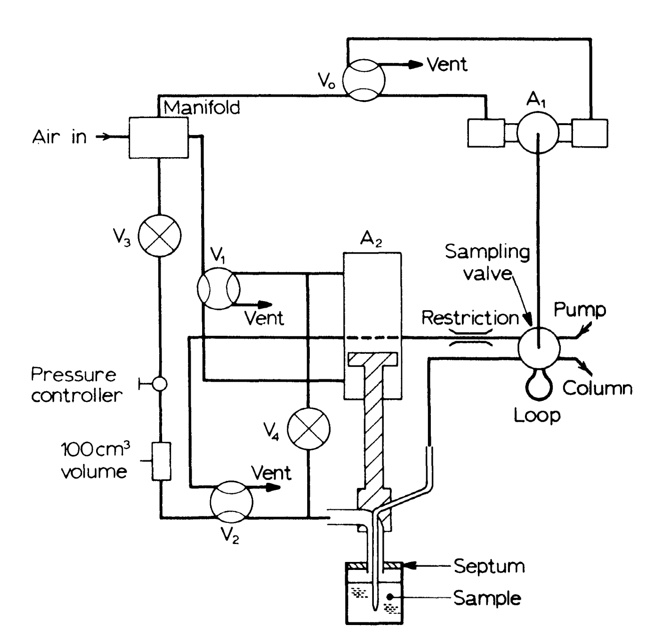

*Figure 2.*

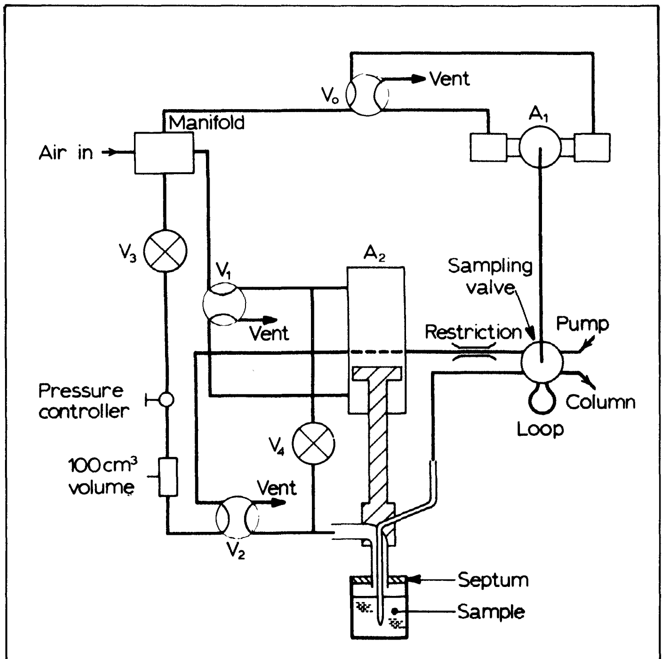

*Figure 2a.*

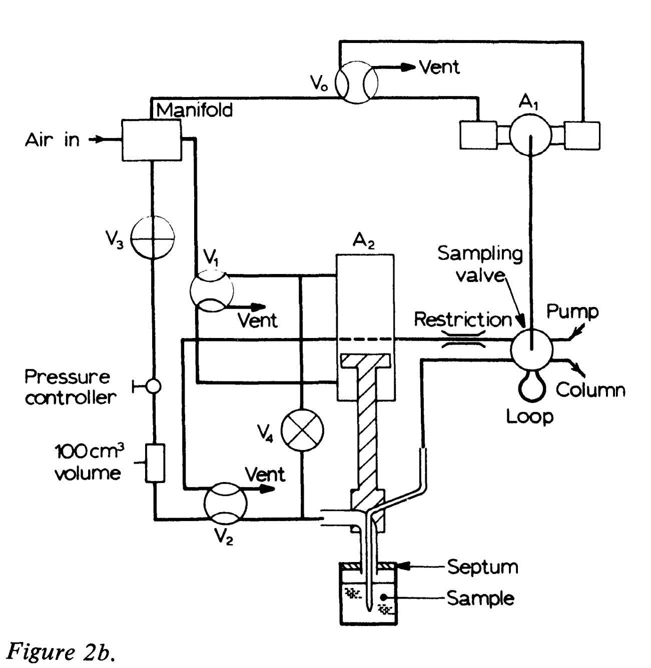

*Figure 2b.*

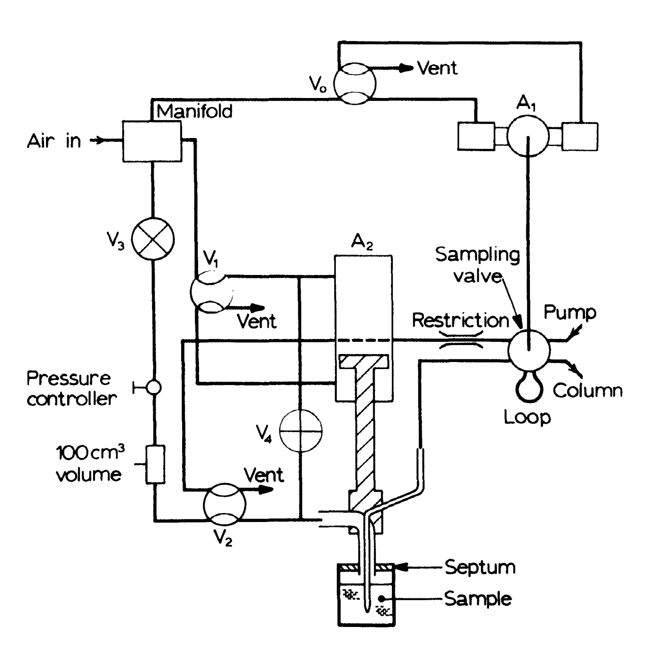

*Figure 2c.*

*Figure 2. Pneumatic layout of autosampler. (a) needle enters vial, (b) the loop is filled and (c) transfer of sample to column.*

| | |
|---|---|
| $A_1$ | *Sampling valve actuator (Rheodyne Inc., type 70.01)* |
| $A_2$ | *Syringe actuator (H. Kuhnke Ltd., type 37291)* |
| $V_0$ | *Spool valve (H. Kuhnke Ltd., type 87-030-01)* |
| $V_1, V_2$ | *Spool valves (H. Kuhnke Ltd., type 44-250-1)* |
| $V_3, V_4$ | *Solenoid valves (H. Kuhnke Ltd., type 65111)* |

taken from a vial without the removal of the needle assembly between samples. When open, it rapidly equalises the pressure in the two lines.

The functions controlled by these valves and their corresponding bit states when not energised (≡ 0) and when energised (≡ 1) are summarised in Table 1.

The autosampler and turntable are operated by an eight-bit control word transmitted from the microcomputer via an interface circuit. Bits 0 to 4 inclusive switch the solenoid valves in the pneumatic control system. Each bit is used to switch a relay (131A-4) which, in turn, switches the corresponding solenoid valve (Bit 0 controls valve $V_0$ etc.). The remaining bits of the control word (5 - 7) are connected to the three inputs of the SAA 1027 integrated circuit used to control the stepper motor. Bit 5, which is connected to the trigger input is pulsed to step the motor. Bit 6 is connected to the set input, an option which is not used in this application. Bit 7 is connected to the input which determines the direction of rotation (1 ≡ anticlockwise, 0 ≡ clockwise).

By transmitting an appropriate timed sequence of control words, any one of a variety of operational modes can be effected; for example, multiple samples can be taken from a single vial or a number of different consecutive samples can be analysed. Optional purge and rinse routines can be called to overcome inter-sample contamination. Table 2 shows a typical control sequence to sample repetitively from a single vial.

### Data handling

### Analogue to digital converter

The signal from the detector (Pye Unicam Ltd. model

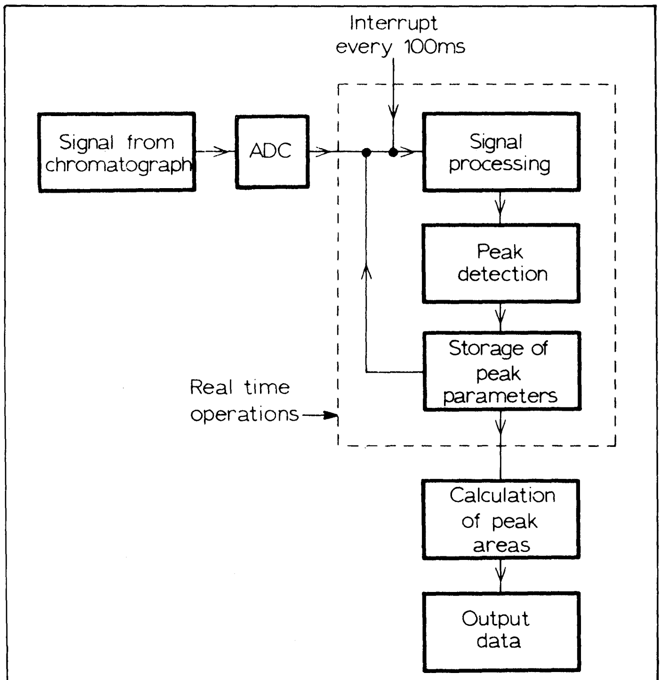

*Figure 3. Block diagram of chromatographic data handling.*

LC3-UV) is digitised using a voltage to frequency converter and 16 bit counter before being processed in the microcomputer. The signal input is buffered using a low noise, low drift amplier which can also provide switchable gains of 1, 10 and 100. The voltage to frequency converter operates at a full scale frequency of 1 MHz for +10 Vinput and has a usable dynamic range of over $10^5$. The analogue input circuitry is completely isolated from the interface to the microcomputer by a high speed optocoupler driven directly from the converter.

The real time clock signal from the microcomputer latches the 16 bit count into a register, resets the counter and gates the data from the register onto the data highway, as two 8 bit bytes, on receipt of the device select signals. The counter continues incrementing until receipt of the next real time clock pulse, when it is again reset.

A row of light emitting diodes on the front panel allows the 16 bits of data to be shown as true binary information or as a baragraph display. The coding is performed by two read only memories and the display is updated at the real time clock frequency.

**Data processing algorithm**

For quantitative chromatography, it is necessary to measure the area of each peak. The algorithms that may be used to calculate the areas of discrete or overlapping peaks generally belong to two classes:

(1) relatively simple empirical methods
(2) more complex methods such as curve fittings [3], [4] that assume the recorded shape or some mathematical model of a peak e.g. Gaussian or Lorentzian.

The choice of algorithm is determined by the response time of the system, the accuracy required, the total available memory size, and the computing power available. In chromatography where the minimum peak width at half height will be $\approx$ 1 second, we are normally confined to the first group of algorithms because of the limited amount of real time processing that can be carried out in the 100 msec intervals between acquisition of data points. Curve fitting techniques only become practical with the aid of hardware arithmetic devices. One of our microcomputers has been subsequently fitted with an arithmetic unit (Advanced Micro Devices, AM 9511) and the hardware can now perform floating point calculations approximately two orders of magnitude faster than is possible by software alone.

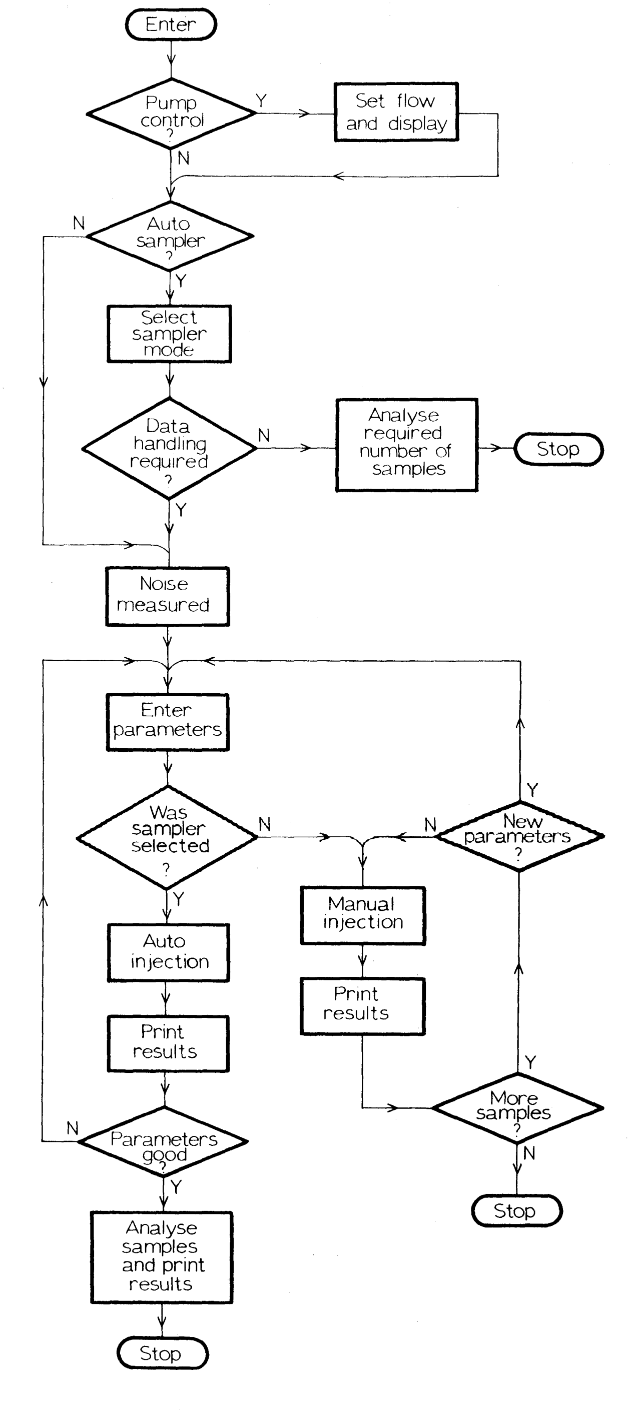

*Figure 4. System operation showing user interaction*

Arithmetic definition of the parameters involved requires a combination of 8 bit, 16 bit and 24 bit integer or floating point (fp) arithmetic. The first two options provide adequate accuracy to define retention times, peak boundaries etc., but are not sufficient for integrated areas where large numbers must be handled. In this instance floating point routines can be incorporated, bearing in mind that a single fp multiplication on the 8080A requires about 5 msec out of the total 100 msec available for real time processing.

The data handling procedure used in this project is shown in Figure 3. Data points are intially summed in order to restrict the number of points across a peak and so optimise

```
**************************************************************************
****                                                                  ****
****            P.R.L. AUTOMATIC LIQUID CHROMATOGRAPH                 ****
****                                                                  ****
**************************************************************************

        REMOTE CONTROL OF PUMP? Y
        FLOW RATE= 2.25
        STABILISATION TIME(MINS)= 5

        AUTOSAMPLER? Y

        TYPE S(SAMPLER),P(PURGE) OR M(MOVE) P

        TYPE S(SAMPLER),P(PURGE) OR M(MOVE) M

        HOW MANY STEPS? +4

        TYPE S(SAMPLER),P(PURGE) OR M(MOVE) S

        THE FOLLOWING ROUTINES ARE AVAILABLE:
        1.SINGLE VIAL,PROBE NOT WITHDRAWN
        2.CONSECUTIVE VIAL SAMPLING
        3.CONSECUTIVE VIAL WITH RINSE
        4.CONSECUTIVE VIAL WITH PURGE
        ROUTINE NUMBER? 1

        DATA HANDLING? Y

        INTERNAL ADC? Y

        SUGGESTED SLOPE SENSITIVITY IS:     3

        DO YOU WISH TO SET ANY PARAMETERS? Y
        SLOPE SENS= 3
        PEAK WIDTH= 8
        DOUBLING TIME(SECS)= 200
        TOTAL TIME(SECS)= 350
        DELAY TIME(SECS)=100
        MINIMUM AREA= 100
        FLOW RATE= 2.25

        PRELIMINARY RUN
```

| PEAK | AREA | RET.TIME | LB | UB | %AREA |
|---|---|---|---|---|---|
| 1 | 29022 | 123.4 | 118 | 128 | 30.04 |
| 2 | 2590 | 142.2 | 136 | 148 | 2.68 |
| 3 | 37659 | 179.3 | 171 | 187 | 38.99 |
| 4 | 27310 | 283.1 | 274 | 292 | 28.27 |

```

        DO YOU WISH TO CHANGE PARAMETERS? N

        HOW MANY SAMPLES? 1

        SAMPLE NO:      1
```

| PEAK | AREA | RET.TIME | LB | UB | %AREA |
|---|---|---|---|---|---|
| -1 | 29031 | 123.3 | 118 | 128 | 30.00 |
| 2 | 2530 | 142.4 | 137 | 149 | 2.61 |
| 3 | 37370 | 179.5 | 171 | 187 | 38.62 |
| 4 | 27822 | 283.7 | 274 | 293 | 28.75 |

```
        END OF RUN.
```

*Figure 5. A listing of the dialogue between computer and user.*

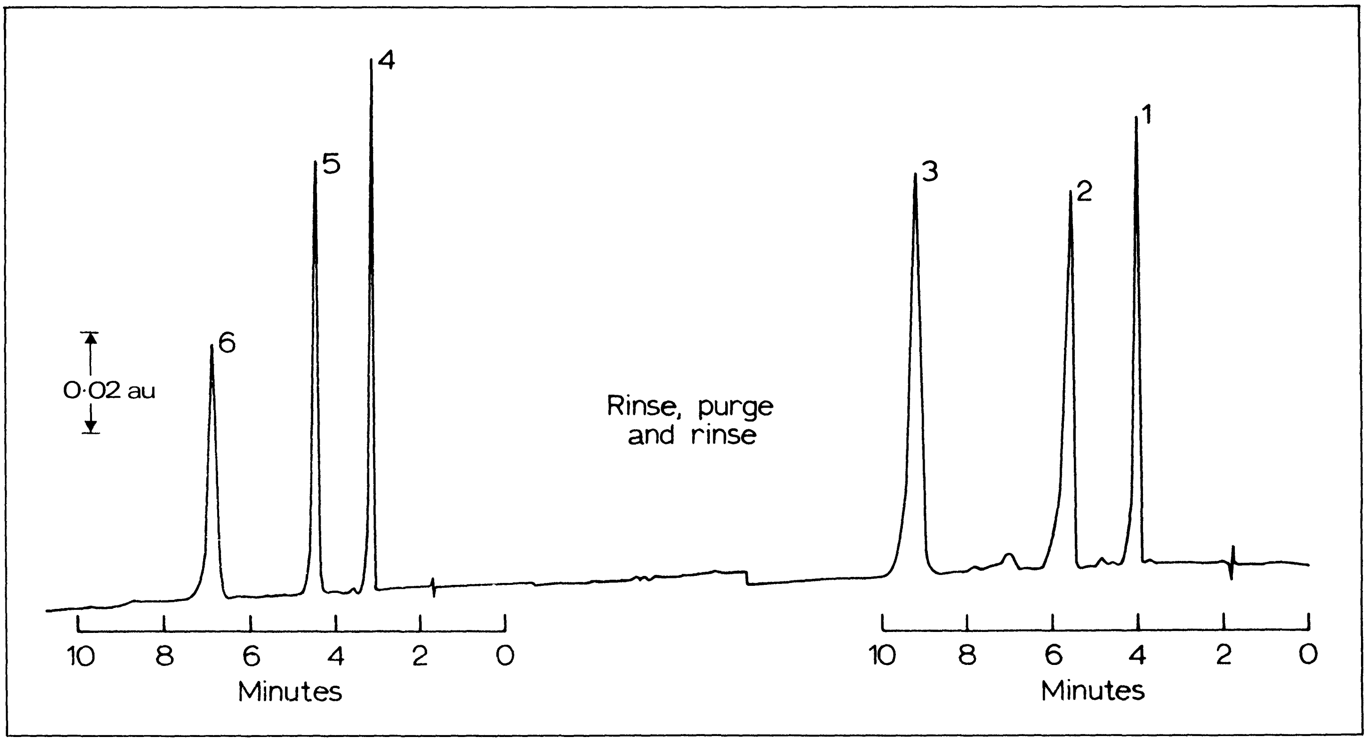

*Figure 6. Consecutive analyses showing the effectiveness of the auto-sampler cleansing routine.*
*Identification: 1 Mesitylene*
*2 Pentamethylbenzene*
*3 Fluoranthene*
*4 Benzene*
*5 Naphthalene*
*6 Anthracene*

the peak width with respect to the subsequent digital differentiation. The signal is then smoothed by a digital filter of the form described by Savitzky and Golay [5], [6]. Peaks are detected by comparison of the first differential of a signal with a preset threshold value. Retention times, boundaries and integrated signals are stored for each peak and output at the end of an experiment after correction for baseline drift and overlap between peaks.

Correct coding of the algorithm was confirmed by loading a series of digital Gaussian curves into the microcomputer to simulate single and grossly overlapping peaks.

The smoothed version of the signal is available at a socket on the microcomputer after conversion to analogue form by the 12 bit DAC.

### Performance

### User interaction

One of our aims when writing the software to control this instrument was to offer the analyst a high degree of interaction and a wide choice of operational modes. The computer 'asks' a series of questions, in plain English, on the VDU to which the user can reply with the appropriate response at the keyboard. This is illustrated by Figure 4 and Figure 5. From the keyboard, the analyst can set the solvent flow rate and can choose the desired sampling mode with or without data handling or, if manual injections are preferred, the data handling procedure can be invoked alone. Before sampling takes place, the detector noise level is measured automatically and the user can then type in various parameters appropriate to the particular analysis in order to obtain the optimum separation and data handling accuracy. After a preliminary analysis, these parameters can be changed if necessary until the optimum conditions have been achieved. The system can then be instructed to proceed with its task without further attention until the required number of samples has been analysed.

### Performance of the auto-sampler

The two most important performance characteristics of the auto-sampler are its precision and the extent to which one sample is affected by residual contamination from the previous sample ('carry-over'). In order to test these characteristics, mixtures of benzene, naphthalene and anthracene in n-hexane were analysed. The separation column (250 mm x 4.8 mm i.d.) was packed with Lichrosorb SI 60 (B.D.H. Ltd.); n-hexane was used as the mobile phase and the wavelength of the UV detector was set to 254 nm. Two modes of automatic sampling were investigated:

(a) repetitive sampling from one vial,
(b) sampling the same mixture contained in consecutive vials.

The initial results in both modes were unsatisfactory; the relative standard deviations of the peak areas obtained were high, especially when sampling from consecutive vials. The answer to these problems was found in simple modifications to the software. An overall improvement was made by the addition of a procedure to pre-wet the transfer tubes with sample before the final sampling operation. The use of a purge before the consecutive vial sampling routine to drive out the residue from the previous sample improved the results for this mode of operation.

After these changes were made, the analysis of ten samples in each mode gave, in both cases, a relative standard deviation of the measured peak areas for all components of the mixture of less than 1.1%. These results represent the precision of the complete chromatograph; it is worth noting that the manufacturers of the sampling valve only claim 'less than 1%' for the valve alone.

As a test of inter-sample contamination, an injection of the hydrocarbon mixture was immediately followed by an injection of n-hexane. The system was not rinsed or purged between these samples. It was found that about 8-10% of the original sample (1420 ppm benzene, 82.0 ppm naphthalene, 3.38 ppm anthracene) was carried over into the second 'blank' injection. This experiment was repeated with the addition of a one-minute purge of each sample line before the n-hexane was sampled. This reduced the carry over almost completely for the more volatile benzene constituent (to 0.2% of the original concentration). There was not sufficient removal of the naphthalene and anthracene however, (to 1.6% and 4.7% respectively).

The addition of a rinse vial containing pure mobile phase was then tried. The complete cycle was, therefore: sample; purge; rinse; purge; blank. With only one rinse between samples, all measurable traces of both benzene and naphthalene were removed but a small amount (< 0.1%) of anthracene was still detected. The analyses of the blank samples were made at maximum sensitivity, eight times more sensitive than for the sample. By a small change in the program, the number of rinses from the same vial was increased to three. This successfully removed all traces of carry-over into the blank sample.

Figure 6 illustrates the results obtained using this combination of purging and multiple rinsing. A blank injection, to demonstrate the cleanliness of the system, was followed by an injection of a mixture of mesitylene, pentamethyl, benzene and fluoranthene. After purging, rinsing and purging again, a second sample, this time the benzene, naphthalene, anthracene mixture, was injected. No trace of contamination by the constituents of the first mixture (which have different elution times to the constituents of the second mixture) can be seen in the second chromatogram.

### Conclusions

The automatic liquid chromatograph described in this paper is a reliable and flexible instrument for both routine and research applications. Its simple, straightforward design and construction, incorporating a high proportion of readily available components, has resulted in low hardware costs, while the flexibility of software control allows a good performance to be achieved.

We have shown that it is capable of repetitive sampling with a precision of 1% relative standard deviation, and effective rinse and purge routines can be readily constructed to ensure that all traces of prior contaminants are removed from the system. The accuracy of the data handling algorithm ensures the integrity of the quantitative results even for those samples which are poorly resolved.

We have demonstrated its use with procedures which would probably satisfy most users; one of the outstanding advantages of software control is, of course, the ease with which procedures can be adapted or supplemented in order to cope with new tasks. This is exemplified by the column switching application [2] which uses a peak detection algorithm developed from the data handling program used in this general purpose liquid chromatograph.

### REFERENCES

[1] Willmott, F.W. and Mackenzie, I. *Analytica Chimica Acta,* (1978) **103,** 401.

[2] Willmott, F.W., Mackenzie, I. and Dolphin, R.J. in Schomburg, G. and Rohrschneider, L. (Editors), "Chromatography 1978", Elsevier, p 151.

[3] Brouwer, G. and Jansen, J.A.J. *Analytical Chemistry,*(1973), **45,** 2239.

[4] Anderson, A.H., Gibb, T.C. and Littlewood, A.B., *Analytical Chemistry,* (1970), **42,** 434.

[5] Savitzky, A. and Golay, M.J.E., *Analytical Chemistry, (1964),* **36,** 1627.

[6] Steiner, J., Termonia, Y. and Deltour, J. *Analytical Chemistry,* (1972) **44,**1906.

### An evaluation of the Kem-O-Mat programmable discrete analyser

**Geoffrey C. Seymour**

*Division of Clinical Chemistry, Northwick Park Hospital, and Clinical Research Centre, Harrow, Middlesex, HA1 3UJ, UK.*

This evaluation extended over a period of three months and follows the recommendations of Broughton *et al.* [1.2].

### The instrument

The Kem-O-Mat (Coulter Electronics Limited*) is a calculator-controlled single channel discrete analyser which may be used for either kinetic or endpoint measurement analyses. The throughput of the instrument varies with the methodology, but can be a maximum of 110 samples/hour, at 37°C, in the kinetic mode and 180 samples/ hour in the endpoint mode.

Results are calculated automatically and are printed in the units of choice. The data are checked before calculation to ensure that linearity limits have not been exceeded and that the initial reagent absorbance was within predetermined limits. The data for non-linear analyses, in the kinetic mode, are printed in absorbance units. The operator has the option to print any or all absorbances since all data are stored in the calculator's memory and can be recalled at the end of the analysis run.

All the key instrument functions are continuously monitored by the calculator and fault warnings are given by an audible alarm and printed message.

There is a comprehensive range of methodologies available for the instrument for which the manufacturer will supply the preprogrammed cassettes and the reagent packs, or suggest a supplier for the latter. The user has the facility to develop his own analytical systems on the instrument and prepare his own programmed cassettes.

### Description

The Kem-O-Mat consists of three modules in the air bath version (Figure 1) and four modules in the water bath version. The air bath version was evaluated in this study. Its three modules are a control module to which is fitted the analyser module while the calculator is connected to the control module by electric cable. The control and analyser modules together occupy 590 x 600 mm of bench space The calculator requires 550 x 365 mm of space. A single 99-132V or 198-264V 50 Hz mains socket supplying 550 VA is the only service required.

The control module contains most of the operational logic, the pump mechanisms and a LED display of the

**Coulter Electronics Ltd., Coldharbour Lane, Harpenden, Herts, UK.*

